# A Link among DNA Replication, Recombination, and Gene Expression Revealed by Genetic and Genomic Analysis of *TEBICHI* Gene of *Arabidopsis thaliana*

**DOI:** 10.1371/journal.pgen.1000613

**Published:** 2009-08-21

**Authors:** Soichi Inagaki, Kenzo Nakamura, Atsushi Morikami

**Affiliations:** 1 Laboratory of Biochemistry, Graduate School of Bio-agricultural Sciences, Nagoya University, Chikusa, Nagoya, Japan; 2 Faculty of Agriculture, Meijo University, Tenpaku, Nagoya, Japan; Iowa State University, United States of America

## Abstract

Spatio-temporal regulation of gene expression during development depends on many factors. Mutations in *Arabidopsis thaliana TEBICHI* (*TEB*) gene encoding putative helicase and DNA polymerase domains-containing protein result in defects in meristem maintenance and correct organ formation, as well as constitutive DNA damage response and a defect in cell cycle progression; but the molecular link between these phenotypes of *teb* mutants is unknown. Here, we show that mutations in the DNA replication checkpoint pathway gene, *ATR*, but not in *ATM* gene, enhance developmental phenotypes of *teb* mutants, although *atr* suppresses cell cycle defect of *teb* mutants. Developmental phenotypes of *teb* mutants are also enhanced by mutations in *RAD51D* and *XRCC2* gene, which are involved in homologous recombination. *teb* and *teb atr* double mutants exhibit defects in adaxial-abaxial polarity of leaves, which is caused in part by the upregulation of *ETTIN* (*ETT*)/*AUXIN RESPONSIVE FACTOR 3* (*ARF3*) and *ARF4* genes. The *Helitron* transposon in the upstream of *ETT*/*ARF3* gene is likely to be involved in the upregulation of *ETT*/*ARF3* in *teb*. Microarray analysis indicated that *teb* and *teb atr* causes preferential upregulation of genes nearby the *Helitron* transposons. Furthermore, interestingly, duplicated genes, especially tandemly arrayed homologous genes, are highly upregulated in *teb* or *teb atr*. We conclude that TEB is required for normal progression of DNA replication and for correct expression of genes during development. Interplay between these two functions and possible mechanism leading to altered expression of specific genes will be discussed.

## Author Summary

DNA replication, repair, and recombination are interrelated processes. Chromatin structure, into which DNA is packaged, is important for regulation of DNA replication, repair, and recombination, as well as gene transcription. After DNA replication and repair, chromatin status including its structure and modification has to be reproduced, and defects in these processes can alter gene expression program because of change in chromatin regulation. Our series of genetic analysis of *tebichi* (*teb*) mutant of model plant *Arabidopsis thaliana* suggest that *TEB* gene is involved in DNA replication and recombination. We also show here that *TEB* gene is required for correct expression of many genes including genes regulating development. From these results we propose that *TEB* gene function is important for maintenance of gene expression pattern after DNA replication and recombination. Furthermore, preferential upregulation of genes near highly duplicated transposons and tandemly arrayed homologous genes are observed in *teb* mutants, suggesting the interrelationship between homologous recombination and gene transcription around the repetitive sequences.

## Introduction

The determination of whether to change or maintain the expression status of groups of genes based on positional information of individual cells is central for the development of multicellular organisms. Because DNA is wrapped around histone octamers to compose nucleosomes, transcriptional regulators and RNA polymerase cannot bind to template DNA and catalyze its transcription without remodeling chromatin to make DNA accessible to those proteins [1]. Epigenetic regulation (such as methylation of cytosine in DNA or histone modification) is increasingly recognized as a normal, essential mechanism to control gene expression at the level of chromatin organization, and thus to regulate many aspects of development or responses to the environment [1]–[4].

Chromatin packaging is also a barrier to processes acting on DNA other than transcription, namely replication, repair and recombination, and thus chromatin structure is remodeled to loosen it during these processes [5]–[8]. To preserve and inherit genetic information, chromatin has to be reassembled and the epigenetic information it carries has to be reestablished after DNA replication and repair. However, because the replication of the genome is regulated in part spatiotemporally, the S phase may offer an opportunity for cells to reprogram genome-wide epigenetic information, leading to a change in gene expression pattern [6],[9]. In contrast, DNA repair is an unscheduled process after DNA damage that occurs at any time and place, potentially activating gene expression in an unregulated manner [5],[6],[10]. DNA damages such as double-strand breaks (DSBs) have been shown to change the local histone modification pattern, which may change epigenetic information (reviewed in [5]).

To investigate the link between DNA damage and chromatin-based gene regulation, the plant *Arabidopsis thaliana* offers an excellent model, because there are a number of mutants affecting both the DNA damage response and chromatin-based gene silencing. The *FASCIATA1* (*FAS1*) and *FAS2* genes of *A. thaliana* respectively encode the large and middle subunits of chromatin assembly factor 1 (CAF-1) [11]. CAF-1 facilitates incorporation of histones H3 and H4 into newly synthesized DNA during DNA replication [12] and repair [13]. Loss-of-function *fas1* and *fas2* mutants have fasciated stems, disrupted leaf phyllotaxy, narrow, dentate leaves, and short roots [14], and show a disrupted expression pattern of developmentally regulated marker genes [11]. *fas* mutants show increased levels of DSBs and highly express DNA damage-inducible genes even under normal growth conditions [15]–[17]. In addition, formation of heterochromatin and transcriptional gene silencing (TGS) are impaired in *fas* mutants [17]–[19]. Although these pleiotropic phenotypes of *fas* mutants are essentially consistent with the idea that FAS reorganizes chromatin and preserves epigenetic information during DNA replication and repair, the cause-effect relationship between these phenotypes and the specificity of the target genes with affected expression have yet to be clarified.

Mutations with defects in MRE11, which is involved in repair of DSBs and DNA damage-associated cell cycle checkpoint control [20], in the RPA2 subunit of replication protein A (RPA), which is a single-stranded DNA binding protein involved in DNA replication and repair [21], and in the small subunits of ribonucleotide reductase (RNR), which is involved in the production of deoxyribonucleotides needed for DNA synthesis, show similar phenotypes to *fas* mutants, including sensitivity to DNA damage and TGS release [19], [22]–[26]. These results suggest that defective DNA synthesis causes DNA damage and aberrant expression of genes in both euchromatin and heterochromatin, possibly through impaired chromatin organization.

Similar phenotypes in development, DNA damage response, and TGS are also observed in mutants impaired in the plant-specific *TONSOKU*/*BRUSHY1*/*MGOUN3* (*TSK*/*BRU1*/*MGO3*) gene [19],[27],[28]. Phenotypic similarities between *tsk*/*bru1*/*mgo3* mutants and *fas*, *mre11*, *rpa2*, and *rnr* mutants, combined with the observation that the *Nicotiana tabacum* homolog of the *TSK*/*BRU1*/*MGO3* gene is predominantly expressed at S phase in synchronously cultured tobacco BY-2 cells suggest that TSK/BRU1/MGO3 protein is involved in the structural and functional maintenance of chromatin during DNA replication [19],[29]. The TSK/BRU1/MGO3 protein has LGN repeats and leucine-rich repeats, both of which are involved in protein-protein interactions [19],[27],[28],[30], and thus may function as a scaffold of proteins involved in DNA replication, repair, and chromatin maintenance.

We previously reported that the *TEBICHI* (*TEB*) gene of *A. thaliana* encodes a protein with both DNA helicase and polymerase domains that are conserved among plants and animals [31]. Its animal homologs (namely, *Drosophila melanogaster* MUS308 and mammalian DNA polymerase θ [POLQ]) have been reported to be involved in tolerance to DNA damage [32],[33], prevention of chromosome breakage [34], and somatic hypermutation of immunoglobulin genes [35]. Loss-of-function *teb* mutations cause various morphological defects, including short roots, abnormal leaf shape and fasciated stems [31]. In addition, *teb* mutants are hypersensitive to DNA damage, constitutively express DNA damage-responsive genes, and accumulate cells expressing a G2/M-specific reporter, *cyclinB1;1:GUS* (*CYCB1;1:GUS*) [31],[36]. However, unlike other mutants exhibiting similar developmental phenotypes and DNA damage response, *teb* mutants do not upregulate a marker of TGS, transcriptionally silent information (TSI). This result suggests that chromatin-based silencing of heterochromatic genes is not impaired in *teb*. However, the phenotypic similarity between *teb* and the preceding mutants suggests that chromatin-based regulation of euchromatic gene expression is affected in *teb*. If so, *teb* mutants may be a good model to explore the relationship between DNA damage, chromatin regulation, and developmental program.

In the present study, we conducted genetic and global gene expression analyses to explore the link between DNA damage responses and developmental phenotypes of *teb* mutants. We found that *TEB* genetically interacts with *ATR*, which is involved in the DNA replication checkpoint, and that expression of a number of tandem and dispersed duplicated genes and genes near *Helitron* transposons is activated in *teb* mutants. Furthermore, we found that the upregulation of two genes near *Helitron* transposons, *ETT*/*ARF3* and *ARF4* genes, in *teb*, plays a role in partial abaxialization of leaves, which is a newly found phenotype of *teb* mutants. We propose a DNA replication-coupled mechanism that maintains the chromatin state of regions around duplicated sequences for correct gene expression during development.

## Results

### *atr* mutations enhance developmental phenotypes of *teb* mutant but suppress accumulation of cells expressing *CYCB1;1:GUS*

**Figure 1:**
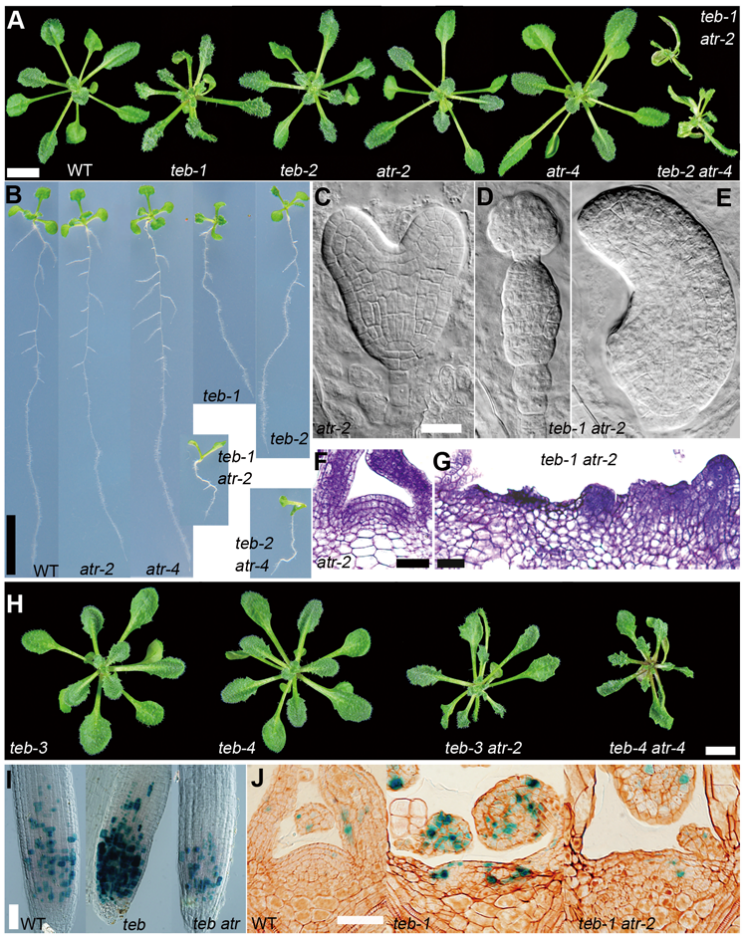
*atr* mutations enhance *teb* mutant phenotypes associated with development but suppress accumulation of cells expressing *CYCB1;1:GUS*. (A) Morphology of shoots from 3-week-old wild-type (WT) and mutant plants. Scale bar, 5 mm. (B) Roots of 12-day-old seedlings. Scale bar, 10 mm. (C–E) Mid-heart embryo from an *atr-2* plant (C), and globular embryo (D) and late-heart embryo (E) from *teb-1 atr-2* plants. Scale bar, 25 µm. (F, G) Shoot apical meristems (SAM) from 11-day-old *atr-2* (F) and *teb-1 atr-2* (G) plants. Scale bars, 50 µm. (H) Shoots of 3-week-old plants of weak *teb* alleles and double mutants for weak *teb* alleles and *atr*. Scale bar, 5 mm. (I, J) GUS-stained root tips (I), and SAMs (J) of *CYCB1;1:GUS*-introduced wild-type (WT), *teb-1*, and *teb-1, atr-2*. Scale bars, 100 µm.

To elucidate the molecular link between DNA damage responses and developmental phenotype in *teb*, we analyzed the genetic interaction of *TEB* with *ATM* and *ATR*. The ATM and ATR protein kinases are key regulators of cell cycle checkpoints conserved among eukaryotes, and are involved in sensing DNA damage and activating downstream regulators of cell cycle progression and DNA repair. ATM is activated primarily by DSBs, whereas ATR is activated when replication forks become stalled (reviewed in [37]). The *A. thaliana* homologs of ATM and ATR function in transcriptional responses after the application of DSB and DNA replication stress, respectively [38],[39]. Although *atm* and *atr* mutants do not show defects in growth and development in the absence of external stress (Figure 1A and 1B, Figure S1; see also [39],[40]), *atm* mutant plants are hypersensitive to DNA-damaging agents, such as g-irradiation, but rather insensitive to replication-blocking agents, such as hydroxyurea or aphidicolin [39], and *atr* mutants are hypersensitive to replication-blocking agents but also mildly sensitive to γ-irradiation [40].

We constructed double mutants of *teb* with *atm* and *atr*, and analyzed their phenotypes. We found that *atr* mutations enhanced the developmental phenotype of *teb*; *teb atr* double mutants exhibited severe growth retardation (Figure 1A), shorter roots than *teb* (Figure 1B), and more severe morphological defects in leaves, shoot apical meristems (SAMs), and embryos than *teb* (Figure 1A, 1D, 1E, and 1G; [31]), whereas *atr* mutants did not show any alteration of morphology in embryos or meristems (Figure 1C and 1F). Furthermore, *atr* also affected the phenotypes of weak alleles of *teb* (*teb-3* and *teb-4*), which by their own do not cause morphological defects (Figure 1H). On the other hand, *atm* did not appear to have any effect on the development-related phenotype of *teb* (Figure S1). These results strongly suggest that the function of TEB is associated with DNA replication.

We next examined the effect of *atr* on accumulation of cells expressing *CYCB1;1:GUS* in *teb*. The accumulation of cells expressing *CYCB1;1:GUS* normally observed in *teb* was largely suppressed by *atr* (Figure 1I and 1J). Aphidicolin-induced accumulation of cells expressing *CYCB1;1:GUS* is suppressed by *atr*, suggesting that ATR is responsible for a cell cycle checkpoint following arrest of DNA replication [40]. Thus, our results suggest that *teb* activates the ATR-mediated DNA replication checkpoint, which is then followed by cell cycle arrest at G2/M. However, the developmental phenotype of *teb* was enhanced rather than ameliorated by *atr* mutation. Taken together, these results suggest that a defect in DNA replication or an event associated with it, rather than the resulting defect in cell cycle progression, is associated with the morphological phenotype of *teb*.

To understand cellular defects leading to the morphological phenotypes of *teb* and *teb atr*, we first examined the extent of cell death using trypan blue staining. DNA damage-induced cell death is well-characterized in animals, and the aphidicolin-treated *atr* mutant of *A. thaliana* shows nuclear degradation, suggesting that an ATR-dependent checkpoint plays a critical role in protecting the genome and preventing cell death [40]. Although *teb atr* and *teb atm* double mutants showed some trypan blue staining, single *teb* mutants were unstained, and the cell death phenotype did not correlate with the severity of the morphological phenotype of these mutants (Figure S2). We concluded that cell death does not play a major role in the morphological phenotype of *teb*.

### *teb* and *teb atr* affect leaf adaxial-abaxial polarity

**Figure 2:**
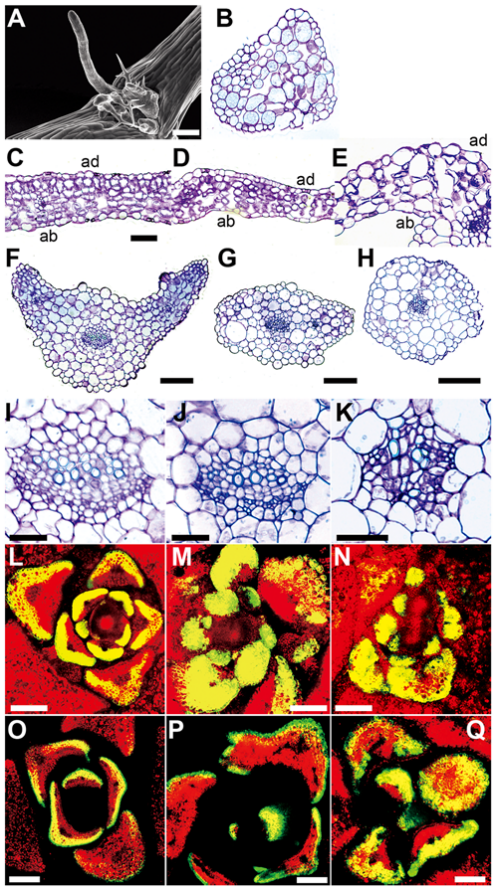
*teb* and *teb atr* are defective in adaxial-abaxial leaf polarity. (A, B) Scanning electron micrograph (A) and transverse section (B) of filamentous leaves of *teb-1 atr-2*. Scale bar for (A), 200 µm. (C–K) Transverse sections of the leaves (C–E), the petioles (F–H), and the vascular bundles (I–K) of wild-type (C, F, I), *teb-1* (D, G, J), and *teb-1 atr-2* (E, H, K). ab, abaxial; ad, adaxial. Scale bars, 50 µm (C–E), 100 µm (F–H), and 25 µm (I–K). (L–Q) Expression of *FILp:GFP* in transverse sections of young leaves in wild-type (L, O), *teb-1* (M, P), and *teb-1 atr-2* (N, Q). Green, GFP; red, chlorophyll autofluorescence; yellow, overlap of these two signals. Scale bars, 100 µm.

In detailed analysis of the phenotype of *teb atr* double mutants, we noticed that *teb atr* plants frequently develop filamentous leaves that are radially symmetrical (Figure 2A and 2B). About half (61/107) of *teb atr* plants developed one or more filamentous leaves. Establishment of a boundary between adaxial (upper) and abaxial (lower) cells is required for the formation of flat leaf blades, and thus a complete loss of adaxial-abaxial polarity leads to formation of radially symmetrical leaves [41]. Therefore, we examined the adaxial-abaxial polarity of leaves in *teb* and *teb atr* mutants. In wild-type leaves, a layer of closely packed palisade cells and loosely packed spongy mesophyll cells reside adaxially and abaxially, respectively (Figure 2C). However, adaxial palisade cells were missing in some regions of *teb* leaves (Figure 2D). Although a number of leaves were not radially symmetrical in *teb atr* plants, the mesophyll tissue consisted largely of spongy mesophyll-like cells in these somewhat expanded leaves of *teb atr* (Figure 2E). Likewise, the polarity of transverse sections of the petioles was also altered in *teb* and *teb atr* (Figure 2F–2H). In addition, polarity of vascular bundles in *teb atr* was also perturbed; development of phloem cells around xylem, in contrast to wild-type, in which xylem and phloem respectively develop adaxially and abaxially, although the vascular polarity of *teb* was almost normal (Figure 2I–2K).

We also analyzed the expression of green fluorescent protein (GFP) under the control of the *FILAMENTOUS FLOWER* (*FIL*) promoter (*FILp:GFP*); expression is observed only in the abaxial region of wild-type leaves (Figure 2L and 2O; [42]). Expression of *FILp:GFP* occurred ectopically in the adaxial regions of some *teb* and *teb atr* leaves (Figure 2M, 2N, 2P, and 2Q). Looking specifically at radially symmetrical leaves from *teb atr* plants, we observed expression of *FILp:GFP* around the outer surface of these leaves (Figure 2Q). Taken together, these results support the stochastic occurrence of partial abaxialization in *teb* and *teb atr* leaves.

### *teb* and *teb atr* upregulate *ETT* and *ARF4* genes

**Figure 3:**
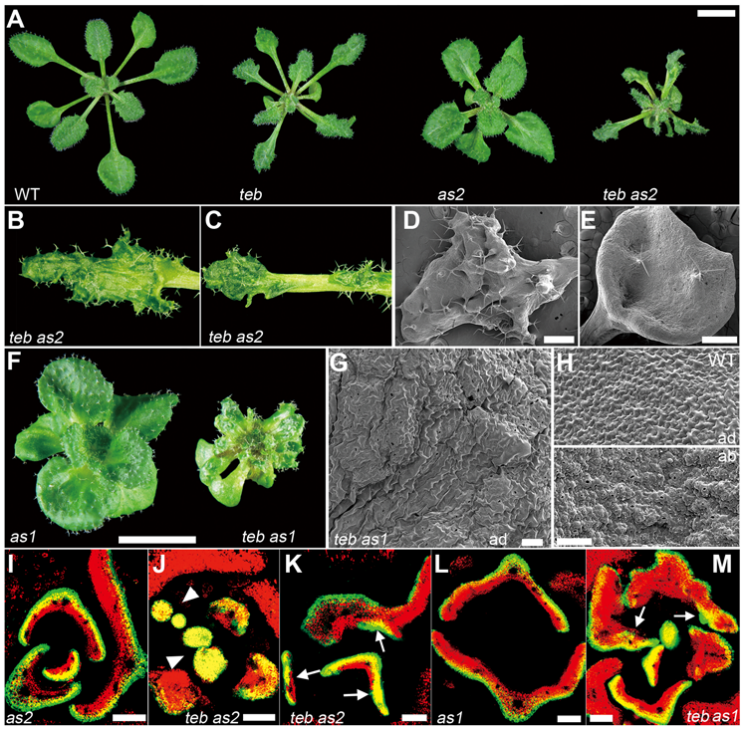
*as1* and *as2* enhance the leaf polarity defect in *teb*. (A) Rosette phenotypes of 3-week-old wild-type (WT), *teb-1*, *as2-1*, and *teb-1 as2-1* plants. Scale bar, 5 mm. (B, C) Adaxial view of the rosette leaves of *teb-1 as2-1*. (D, E) Scanning electron microscopy images of adaxial side of *teb-1 as2- 1* leaves. A number of protrusions from the adaxial surface (*D*) and a trumpet-shaped leaf (E). Scale bars, 500 µm. (F) Rosette phenotypes of 3-week-old *as1-1* and *teb-1 as1-1* plants. Scale bar, 5 mm. (G, H) The epidermal surface of adaxial side of a *teb-1 as1-1* leaf (G), and adaxial (ad; top) and abaxial (ab; bottom) sides of wild-type leaves (H). Scale bars, 100 µm. (I–M) Expression of *FILp:GFP* in transverse sections of young leaves in *as2-1* (I), *teb-1 as2-1* (J, K), *as1-1* (L), and *teb-1 as1-1* (M). Green, GFP; red, chlorophyll; yellow, overlap of these two signals. Arrowheads indicate leaves which express *FILp:GFP* throughout the leaves and show no sign of adaxial-abaxial polarity, and arrows indicate ectopic expression of *FILp:GFP* in the adaxial domain of leaves. Scale bars, 100 µm.

We analyzed the adaxial-abaxial polarity phenotype of *teb* and *teb atr* in more detail to elucidate the relationship between the molecular function of *TEB* and the developmental phenotype of *teb* mutants. In recent years, molecular factors that are involved in establishment of leaf adaxial-abaxial polarity have been identified (reviewed in [43]). We generated a series of double mutants combining *teb* with mutations in regulatory genes involved in adaxial-abaxial polarity. Mutants affected in genes such as *REVOLUTA* (*REV*), *PHABULOSA* (*PHB*), *KANADI1* (*KAN1*), and *FIL* did not appear to enhance or suppress the *teb* phenotype (data not shown). However, *asymmetric leaves 1* (*as1*) and *as2* mutations affected the leaf phenotype of *teb* (Figure 3). *teb as2* double mutant plants exhibited leaves with several lobes and a very ruffled surface, in addition to some trumpet-shaped leaves (Figure 3A–3E), indicating severe defects in adaxial-abaxial polarity. Likewise, *teb as1* double mutant plants showed a severe defect in leaf expansion (Figure 3F). In addition, the epidermal surface of the adaxial side of *teb as1* leaves showed an undulating surface with a high density of stomata, resembling the abaxial leaf surface of wild-type, rather than the adaxial surface, which is flat and has a low density of stomata (Figure 3G and 3H). Moreover, *teb as1* and *teb as2* exhibited higher ectopic expression of *FILp:GFP* in the adaxial domain of leaves compared with the *teb* single mutant (Figure 3I–3M).

**Figure 4:**
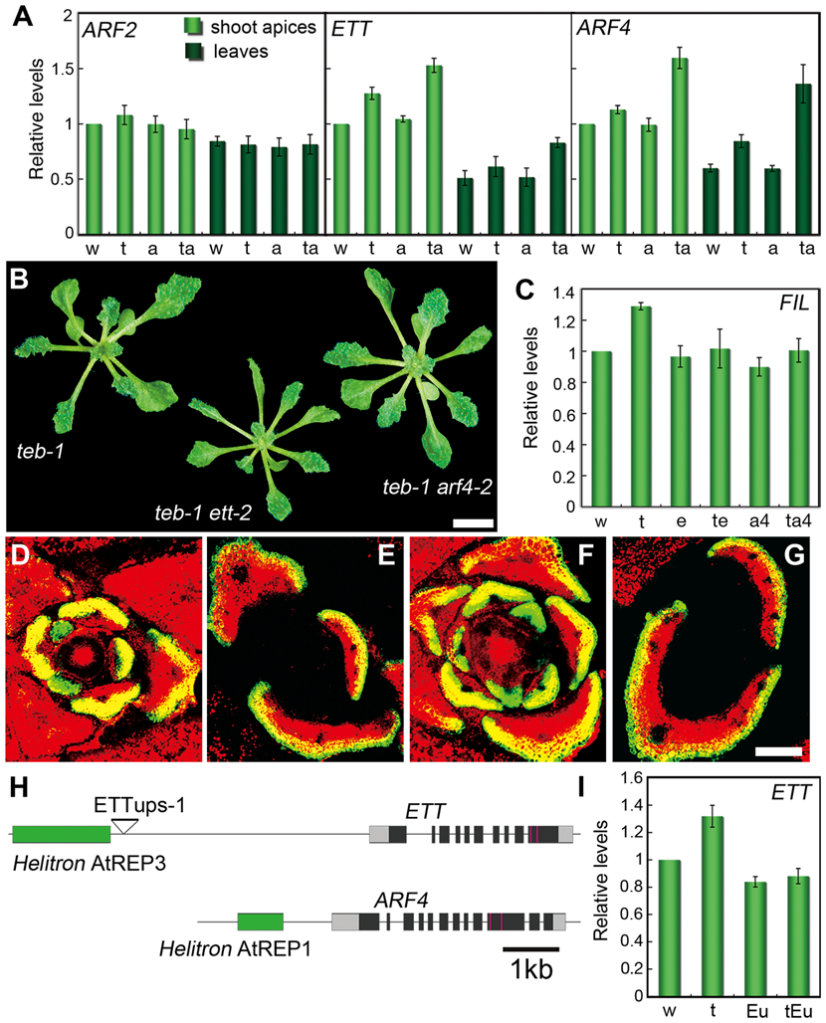
TEB and ATR regulate the expression of *ETT* and *ARF4*. (A) The levels of *ARF2*, *ETT*, and *ARF4* mRNAs in shoot apices and leaves of the wild-type (w), *teb-1* (t), *atr-2* (a), and *teb-1 atr-2* (ta), as determined by quantitative real time RT-PCR. The values are expressed as the ratio of the value obtained for the specific sample to the value obtained for the shoot apices of wild-type. The values shown are the means of 5 biological replicates ±S.E. (B) Rosette phenotypes of 3-week-old *teb-1*, *teb-1 ett-2*, and *teb-1 arf4-2* plants. Scale bar, 5 mm. (C) The levels of *FIL* mRNA in the shoot apices of *teb-1* (t), *ett-2* (e), *teb-1 ett-2* (te), *arf4-2* (a4), and *teb arf4-2* (ta4) relative to wild-type (w). The values shown are the means of 4 biological replicates ±S.E. (D–G) Expression of *FILp:GFP* in transverse sections of young leaves in *teb-1 ett-2* (D, E) and *teb-1 arf4-2* (F, G). Scale bar, 100 µm. (H) Diagram of the genomic regions around the *ETT* and *ARF4* loci. Green, *Helitron* insertions; dark gray, coding regions; light gray, 5′- and 3′-untranslated regions. Red, target sites for *tasiR-ARF*. Triangle, a T-DNA insertion site in ETTups-1. (I) The levels of *ETT* mRNA in the shoot apices of *teb-1* (t), ETTups-1 (Eu), and *teb-1* ETTups-1 (tEu) relative to wild-type (w). The values represent the means from 3 experiments with 2 separate seed pools (6 sets of data) ±S.E.

The leaves of *teb as1* and *teb as2* resemble leaves of double mutants of *as1* or *as2* in combination with genes encoding components of the *trans*-acting short-interfering RNA (ta-siRNA) pathway [44]–[46]. One ta-siRNA, *tasiR-ARF*, targets the mRNAs of three *AUXIN RESPONSE FACTOR* (*ARF*) genes, *ARF2*, *ETTIN* (*ETT*)/*ARF3* (hereafter *ETT*), and *ARF4*, for cleavage, and *ETT* and *ARF4* are overexpressed in mutants defective in the ta-siRNA pathway [47]–[49]. *ETT* and *ARF4* have also been reported to redundantly specify abaxial cell fate [50]. Thus, we examined the expression of *ARF2*, *ETT*, and *ARF4* in *teb* and *teb atr*. We found a small but reproducible increase in the expression of *ETT* and *ARF4*, but not of *ARF2*, in shoot apices and leaves of *teb* plants, and the effect was enhanced by *atr* (Figure 4A).

To examine the effect of increased expression of *ETT* and *ARF4* on the phenotype of *teb*, we analyzed *teb ett* and *teb arf4* mutants. *ett* and *arf4* had an insignificant effect on the overall leaf phenotype of *teb* (Figure 4B). However, the increased and ectopic expression of *FIL* in *teb* was largely suppressed by the *ett* and *arf4* mutations (Figure 4C–4G), suggesting that upregulation of *ETT* and *ARF4* plays a role in leaf abaxialization associated with the ectopic expression of *FIL* in *teb* mutants. Since overexpression of *ETT* and *ARF4* alone does not cause any defect in adaxial-abaxial polarity or cause ectopic expression of *FIL* in mutants affected in the ta-siRNA pathway [44]–[46], abnormal expression of some other gene is probably responsible for the leaf polarity defect of *teb*. Thus, we concluded that the abaxialization of the leaves in *teb* is caused at least in part by increased expression of *ETT* and *ARF4*.

We next analyzed genetic interactions between *TEB* and *ARGONOUTE7* (*AGO7*) or *RNA-DEPENDENT RNA POLYMERASE6* (*RDR6*), which encode components of the ta-siRNA pathway. *ago7* and *rdr6* slightly exaggerated the phenotype of *teb* leaves. Additionally, *ETT* and *ARF4* were expressed at higher levels in *teb ago7* and *teb rdr6* compared with *ago7* or *rdr6* (Figure S3). This additive effect of *teb* and *ago7* or *rdr6* on the expression of the *ETT* and *ARF4* genes suggests that TEB regulates expression of *ETT* and *ARF4* by a pathway different from the ta-siRNA pathway.

### Upregulation of genes near *Helitron* transposons in *teb* and *teb atr*

A survey of the genomic sequence around *ETT* and *ARF4* revealed the presence of *Helitron*-like sequences upstream of both genes (Figure 4H). *Helitrons* are a class of DNA transposons recently discovered in a number of eukaryotes, and they and their nonautonomous derivatives constitute more than 2% of the *A. thaliana* genome [51]. The *Helitron*-like sequences upstream of *ETT* and *ARF4* are nonautonomous elements designated AtREP3 and AtREP1, respectively [51]. To determine whether *Helitron* elements play a role in upregulation of nearby genes in *teb* mutants, we looked at the effect of a T-DNA insertion (ETTups-1) between the *Helitron* element AtREP3 and the *ETT* locus on the expression of *ETT* in *teb* (Figure 4H). Plants with both the *teb-1* mutation and the ETTups-1 insertion expressed *ETT* at the same level as ETTups-1 plants, which is lower than the level in *teb* (Figure 4I). It would appear that ETTups-1 increases the distance between AtREP3 and the *ETT* gene, and neutralizes the effect of AtREP3 on the expression of *ETT* in *teb*. Since plants harboring only ETTups-1 did not show any defect in leaf morphology (data not shown), ETTups-1 probably does not have much of an impact on the normal expression pattern of *ETT* in wild-type, suggesting that *Helitron* AtREP3 does not have a major role in the normal expression of *ETT*. These results suggest that upregulation of *ETT* in *teb* may be linked to the presence of an upstream *Helitron*, although the involvement of the other upregulating element around ETTup-1 insertion cannot be excluded. To support this result, we examined the expression of randomly chosen 4 genes with *Helitron* AtREP3 in their upstream regions. As a result, we found a small but reproducible increase in the expression of these 4 genes in *teb* plants, and the effect was enhanced by *atr* (Figure S4A).

**Figure 5:**
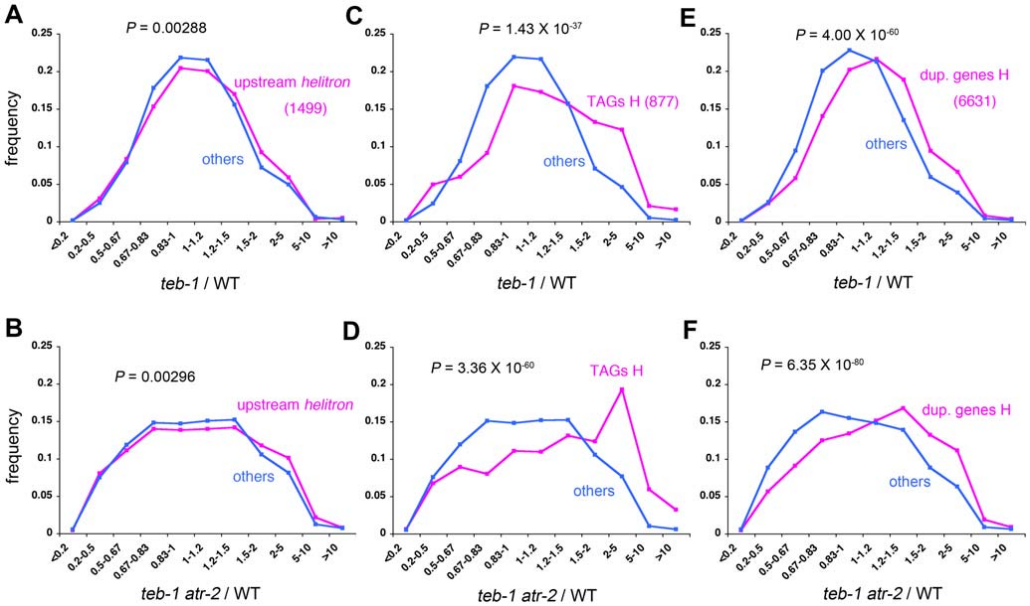
Upregulation of genes close to *Helitron* transposons and of TAGs in *teb* and *teb atr*. Frequency distribution histograms of the ratio of expression in *teb-1* (A, C, E) or *teb-1 atr-2* (B, D, F) to wild-type (WT) for all genes with ‘P’ or ‘M’ call for at least 2 of 4 samples in first experiment. Genes with *Helitrons* transposons in their upstream 2 kb regions (magenta lines in A, B), TAGs (magenta lines in C, D), and duplicated (dup.) genes (magenta lines in E, F) are shown. *P*-values for differences of ratios between two gene groups were calculated using the Student t tests. H, high stringency homology definition (see Materials and Methods).

We next analyzed global gene expression using a microarray approach (Figure S5) to see whether the effect of the *teb* mutation on the expression of genes having a nearby *Helitron* insertion is a general one. We examined the expression of a set of the genes with *Helitron* elements of more than 300 bp in their upstream 2 kb regions, in our microarray experiments. We found that genes with upstream *Helitron* elements showed weak but statistically significant tendency to be upregulated in *teb* and *teb atr* (Figure 5A and 5B, Figure S6A, S6B). However, the insertion of *Helitron* elements in nearby regions was not sufficient for upregulation in *teb*, suggesting the involvement of other factors in the upregulation.

### Upregulation of tandem and dispersed duplicated genes in *teb* and *teb atr*

**Figure 6:**
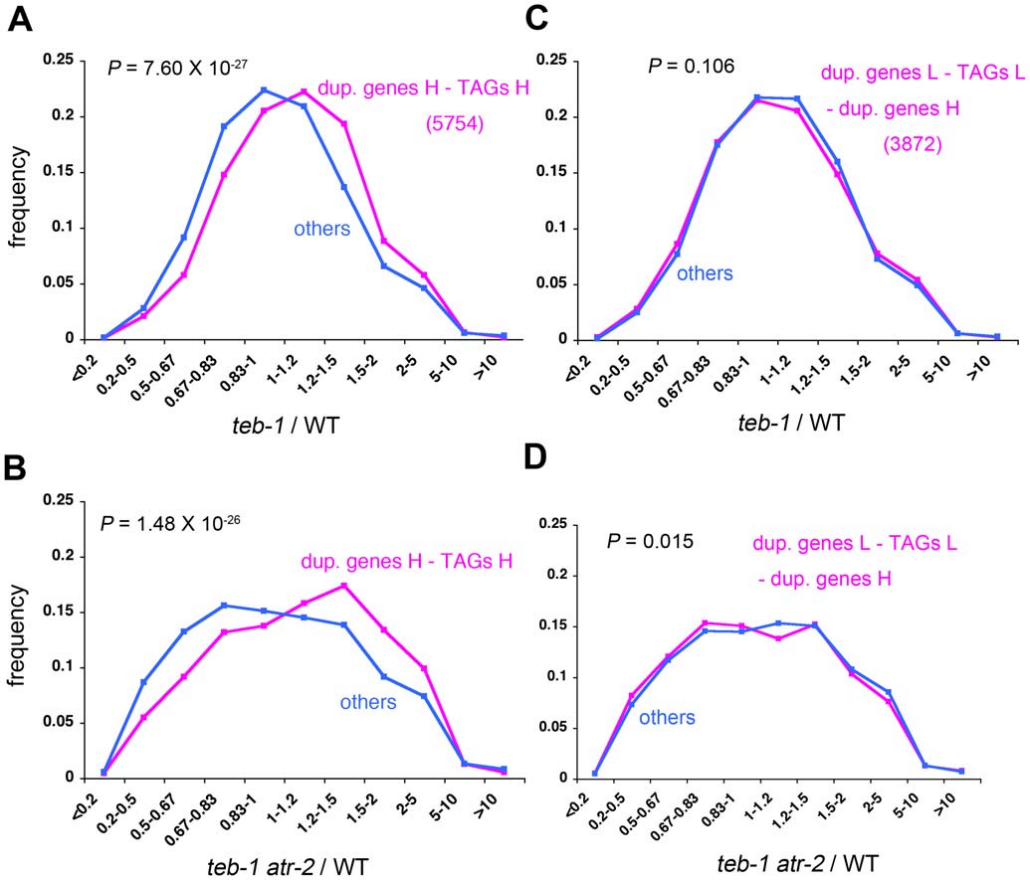
Proximity and homology between genes are important for upregulation of duplicate genes in *teb* and *teb atr*. Frequency distribution histograms of the ratio of expression in *teb-1* (A, C) or *teb-1 atr-2* (B, D) to wild-type (WT) for all genes with ‘P’ or ‘M’ call for at least 2 of 4 samples in first experiment. A group of duplicate genes not tandemly arrayed (dup. genes H – TAGs H) (magenta lines in A, B), and a group of duplicate genes not tandemly arrayed that exhibit a lower level of sequence homology to other genes (dup. genes L – TAGs L – dup. genes H) (magenta lines in C, D) are shown. L, low stringency homology definition; H, high stringency homology definition (see Materials and Methods). Indications of the graphs and the *P*-values denote same things as Figure 5.

Interestingly, we found that many tandemly arrayed homologous genes (TAGs; [52]) are markedly upregulated in *teb* and *teb atr* compared to the wild-type (Figure 5C and 5D, Figure S6C, S6D). We also observed significant increases of expression of duplicated genes, i.e., those with one or more closely related genes somewhere in the genome, in *teb* and *teb atr* (Figure 5E and 5F, Figure S6E, S6F). Because duplicated genes include both TAGs and dispersed duplicated genes, in order to ask whether the upregulation of duplicated genes is solely attributable to the upregulation of TAGs, we first subtracted TAGs from the list of duplicated genes and then again asked whether duplicated genes are upregulated in *teb* and *teb atr*. Tendency of upregulation of these duplicated genes was still observed (Figure 6A and 6B, Figure S6G and S6H). However, this tendency was not observed for non-TAG duplicated genes with low homology to other genes (Figure 6C and 6D, Figure S6I, S6J). These results suggest that duplicated genes are preferentially upregulated in *teb* and *teb atr*, and that both the proximity and the homology between duplicated genes are important factors in upregulation in *teb* and *teb atr*.

Furthermore, we found that the expression of many γ-irradiation-inducible genes [38] was upregulated in *teb* (Figure S7). This result reinforces our previous observations with selected DNA damage-inducible genes [31]. These genes were also upregulated in *teb atr* (Figure S7E, S7F), and to a greater degree than in *teb* (Figure S7G, S7H).

### Genetic interaction between *TEB* and genes involved in homologous recombination

**Figure 7:**
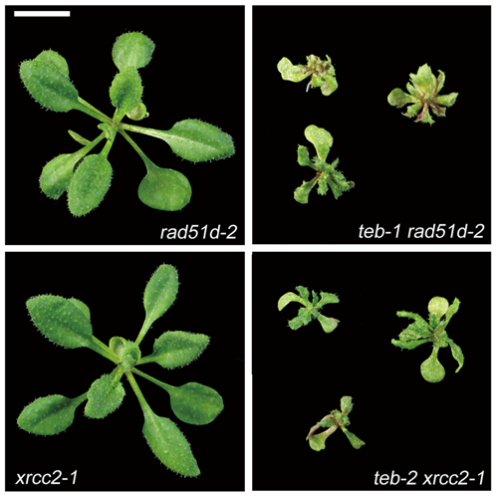
*rad51d* and *xrcc2* mutations enhance the developmental phenotypes of *teb*. Shoot morphology of 3-week-old *rad51d* and *xrcc2*, and *teb rad51d* and *teb xrcc2* double mutant plants. Scale bar, 5 mm.

Recently, it was reported that the recombination-related RAD51D protein is involved in a transcriptional activation of *pathogenesis-related* (*PR*) genes in a *suppressor of npr1 inducible 1* (*sni1*) mutant background of *A. thaliana* [53] (See below). Exploration of reported microarray data of *sni1* [54] revealed that TAGs tend to be upregulated in *sni1* (Figure S8), which is similar to what we observed in *teb* (Figure 5), suggesting *sni1* mutation affects the transcription of TAGs via the function of RAD51D. Furthermore, our microarray data showed that *teb* and *teb atr* upregulate the expression of *PR* genes as in the *sni1* mutant (Figure S9). These results suggest that the global gene expression patterns are similar in *teb* and *teb atr*, and *sni1*. Accordingly, we examined genetic interaction between *TEB* and two recombination-related genes, *RAD51D* and *XRCC2* [55]. As a result, both of *rad51d* and *xrcc2* mutations markedly enhanced the developmental defects of *teb*, whereas *rad51d* and *xrcc2* single mutants did not show any developmental defects (Figure 7).

## Discussion

### Function of TEB in DNA replication and recombination

Here, we demonstrated that *TEB* genetically interacts with *ATR* for developmental phenotypes, cell death, and altered gene expression. Our results provide genetic evidence for a function of TEB in DNA replication to correctly propagate genetic information. The increased expression of γ-irradiation-inducible genes in *teb* and further upregulation in *teb atr* suggest that TEB and ATR prevent the formation or accumulation of DSBs or other types of DNA damage during DNA replication. The mammalian ATR and its yeast homologs, Mec1 and Rad3, are essential for cell survival and are known to be involved in preventing replication fork collapse, DNA breakage, or genome rearrangement, after a stall in the progression of the replication fork, even in the absence of exogenous stresses [56]. However, *atr* mutants of *A. thaliana* are viable and develop normally in the absence of treatment with DNA replication-blocking agents [40]. Hence, *A. thaliana* may have fewer endogenous stresses that perturb DNA replication under normal growth conditions. Otherwise, other proteins may ensure smooth progression of replication forks. We found here that in the presence of the *teb* mutation, an effect of the loss of ATR became apparent, suggesting that TEB has a crucial role in normal progression of DNA replication. In the *teb* single mutant, it is probable that the ATR pathway functions to alleviate the defect in DNA replication by activating any bypass pathway and/or delaying replication and cell cycle progression. Indeed, the accumulation of cells expressing *CYCB1;1:GUS* in *teb* was *ATR*-dependent, suggesting that an ATR-dependent cell cycle checkpoint is activated to delay G2/M progression in *teb*.

Homologous recombination is thought to be important for recovery from stresses that perturb replication, such as DNA damage, nucleotide depletion, or the presence of a specific sequence that hinders progression of a replication fork [57]. Strong genetic interaction between *TEB*, and *ATR* and recombination-related *RAD51D* and *XRCC2* suggest the involvement of TEB in homologous recombination or functionally connected other process during DNA replication. Because double mutants between *teb* and *atr*, *rad51d*, and *xrcc2* are not lethal despite the severe growth retardation, it would be interesting to examine what occurs in the genomic sequences of these double mutants, and how they complete DNA replication.

### Function of TEB in gene expression

Phenotypic overlap between mutants for *TEB*, *FAS*, *MRE11*, *RPA2*, *RNR*, and *TSK*/*BRU1*/*MGO3* suggests functional overlap of these genes in maintenance of chromatin and correct gene expression following DNA replication. Unlike other mutants, however, *teb* did not affect TGS of heterochromatic genes [31], suggesting TEB does not have a major function in the maintenance of heterochromatin. However, we showed here that *teb* affects expression of many genes. Thus, it is possible that TEB regulates the expression of euchromatic genes through chromatin-based manner. In support of the idea that TEB has a role in maintenance of chromatin, we could not identify any double homozygotes for *teb* and *fas2* in the progeny of plants homozygous for *fas2* and heterozygous for *teb* (our unpublished results), suggesting that TEB and CAF-1 have complementary functions in the maintenance of chromatin.

It is interesting that *teb* influenced the expression of a number of genes that do not seem to be directly involved in cellular responses to DNA damage, including tandem and dispersed duplicated genes and genes near *Helitron* transposons. It has been shown in yeast and animal that DSBs or other DNA damages induce local nucleosome depletion and changes in histone modification to make damaged DNA accessible to repair proteins, an effect that also has the potential to impose changes in gene expression [5],[6]. Since TEB seems to function to prevent the formation or accumulation of DNA damage, selective upregulation of TAGs and genes with nearby *Helitron* insertions in *teb* indicates that *teb* affects the chromatin state of these loci due to accumulation of DNA damage in their vicinity.

Taken together, we hypothesize that *teb* affects the chromatin state of regions around tandem and dispersed homologous genes or transposons through unsuccessful homologous recombination and resulting DNA damage during DNA replication. Tandem and dispersed homologous sequences can be the targets of ectopic homologous recombination [58]–[60]. *Helitron* elements are abundant in the genome, the elements are typically large, and the elements share high sequence homology with one another, which seem to increase the chance of ectopic homologous recombination between elements [51],[61]. Indeed, AtREP3 and AtREP1 near *ETT* and *ARF4* genes, respectively, are two of most abundant classes of non-autonomous *Helitrons* [51], and homology search analysis for each of these AtREP3 and AtREP1 against *A. thaliana* genome sequence identified more than a hundred of homologous elements with more than 80% sequence identity entirely or partly. Furthermore, genes having *Helitron* elements of less than 300 bp long in their upstream regions did not show tendency to be upregulated in *teb* and *teb atr* (data not shown), as opposed to genes with upstream *Helitron* of more than 300 bp long (Figure 5). The results that the proximity and the homology between duplicated genes are critical factors for upregulation in *teb* and *teb atr* (Figure 6) also support our hypothesis, because proximity and high degree of homology between repeats increase the frequency of recombination between them [62]–[64].

What mechanism would lead to an altered chromatin state in these specific regions in *teb*? One possibility is that TEB is involved in homologous recombination between repeats, which is activated by a stalled replication fork. Aberrant recombination between repeats in *teb* mutants might result in DNA damage and chromatin disorganization. If so, however, many cells should undergo recombination events between these repeats in wild-type plants, because changes in expression of TAGs are generally large and thus large population of cells should increase their expression in *teb* mutants. This would mean that the DNA sequences of these regions would likely change rapidly even in a single generation, which is unlikely. Alternatively, TEB may repress homologous recombination between repeats by ensuring allelic recombination. *teb* did not show increased recombination between two tandemly arrayed overlapping parts of a GUS transgene [31]. Therefore, it is possible that the initiation of recombination between repeats is triggered by a failure of allelic recombination in *teb*, but *teb* cannot normally undergo recombination between repeats.

It would be interesting to explore possible involvement of specific epigenetic marks in the *teb*-mediated upregulation of *Helitron*-flanked and duplicated genes. At the *A. thaliana recognition of Peronospora parasitica 5* (*RPP5*) locus, comprised of seven duplicated genes, small RNA species corresponding to genic regions are detected [65] and a considerable amount of cytosine methylation was detected in genome-wide mapping study [66]. Another cluster composed of nine chitinase/glycosylase-18 genes is associated with TERMINAL FLOWER 2/LIKE HETEROCHROMATIN PROTEIN 1 (TFL2/LHP1), indicating the association of this locus with histone H3 trimethylation at lysine 27 [67]. These epigenetic marks might regulate the coordinate expression of genes in a cluster. However, in the region around the duplicated genes upregulated in *teb*, we did not find any significant amount of small RNA or cytosine methylation in public databases (http://asrp.cgrb.oregonstate.edu and http://epigenomics.mcdb.ucla.edu/DNAmeth/project.html). In addition, high level of cytosine methylation and small RNAs were found in *Helitron* regions according to these databases. However we did not find any difference in cytosine methylation level in AtREP3 and AtREP1 in the upstream of *ETT* and *ARF4* genes, respectively, between wild-type and *teb* (data not shown).

### Possible interplay between recombination and gene expression

Our knowledge about the interplay between recombination and gene expression is scarce. However, the findings that *sni1* show upregulation of the expression of many TAGs (Figure S8) and RAD51D protein is required for upregulation of *PR* genes in the *sni1* mutant [53] suggest the occurrence of recombination-coupled regulation of gene expression. A large family of resistance (R) genes responsible for recognition of specific pathogenic signals form clusters in the plant genome, and these R genes are subjected to ectopic recombination within or between clusters [60],[68]. Hence, SNI1 and RAD51D may antagonistically control the transcription of R genes in a recombination-coupled manner. *PR* genes themselves also have homologous genes nearby, and *teb* and *teb atr* also upregulate the expression of *PR* genes (Figure S9), suggesting the possibility of direct role of TEB, SNI1, and RAD51D in regulating the expression of *PR* genes. In any case, our observation that the mutations in recombination-related genes enhanced the phenotypes of *teb* supports our hypothesis that there is a genome-wide recombination-coupled maintenance mechanism of chromatin around duplicated sequences. Identification of additional factors involved in the regulation of duplicated genes, analyses of their genetic and physical interactions, and their impact on genetic and epigenetic contexts of the genome will help understand the interplay between recombination and gene expression.

In more general, our results raise the possibility that (tandemly) duplicated genes and *Helitrons* elements play a role in changing expression pattern of genes, in addition to genetic change by recombination and transposition, in the evolutionary process. It has been shown that *Helitrons* are involved in creation of new genes by capturing a part or whole of genes and transposing with them in maize [69],[70]. Tandemly duplicated genes are believed to have a role in genome evolution by homologous crossing over and gene conversion [58]. Our results propose an unidentified potential of these genetic elements to produce expressional and developmental variation.

## Materials and Methods

### Plant materials and growth condition

The strain of *Arabidopsis thaliana* (L.) Heynh. used as “wild-type” in this study was Columbia-0 (Col-0). The *teb* mutants [31] and *CYCB1;1:GUS* plants [36] have been described previously. *atm-2* [39], *atm-4* (SALK_036940), *atr-2* [40], *atr-4* (SALK_054383), *ago7-1* [71], *rdr6-11* [48], *ett-2* [72], *arf4-2* [50], *xrcc2-1* [55], *rad51d-2* (CS830262), and ETTups-1 (SALK_053636) seeds were obtained from the Arabidopsis Biological Resource Center. SALK seeds were generated by the Salk Institute Genomic Analysis Laboratory [73]. Seeds of *FILp:GFP* plants (Watanabe and Okada, 2003) were a gift from K. Okada. Seeds of *as1-1* and *as2-1* mutants [74] were a gift from Y. Machida and Y. Ueno. All mutants and transgenic plants were in the Col-0 genetic background, except for *ett-2*, which was in the Wassilewskija (Ws) background. Plants were grown as described previously [31].

### Genetic analyses

Plants carrying multiple mutations or transgenes were generated by standard genetic crosses and were identified in F2 progeny by phenotypic and genotypic observation. The presence or absence of T-DNA inserts was examined by PCR using an oligonucleotide primer that recognizes the left border of the T-DNA element, PL11: 5′-TTTCGCCTGCTGGGGCAAACCAG-3′, and primers that recognize genomic regions upstream or downstream of the T-DNA of interest, as follows: atm-2F: 5′-CTGTTGAAAGAATGGAAACACAGTAAAG-3′, atm-2R: 5′-GCTCTGCTGCAAGCTTTTTATCC-3′, atm-4F: 5′-GGACTGAAGTACATAAGCTTTTCC-3′, atm-4R: 5′-CTCTGAAAGTTTGTGCAGATGG-3′, atr-2F: 5′-CATCAACAGCTACCATAACTTCAGC-3′, atr-2R: 5′-GCTACGGAGAAAAGTTGCGAAAG-3′, atr-4F: 5′-TATCTGTCTCAGGTGTATCAGCAATG-3′, atr-4R: 5′-TCACTCACAGATTGGTTCTGACAACC-3′, ago7-1F: 5′-CTCTCTATTGGTACTGATTTACTTGC-3′, ago7-1R: 5′-TGCTGCTTCTTCTATTGCTATGGATC-3′, arf4-2F: 5′-AATCCAGTTCTTGTGTCGAGTAGAGTC-3′, arf4-2R: 5′-TTGCAAGACCCTTGGAAACCTATCCAG-3′, xrcc2-1F: 5′-GTTAGAGAGTTTGAGGAACTTTGAG-3′, xrcc2-1R: 5′-GAGATGAAGCGACTATAGCAAC-3′, rad51d-2F: 5′-GCATTGCTCAATTTATCTGCTCC-3′, rad51d-2R: 5′-GCTTAATACCTGCAACCTCAAAG-3′, ETTups-1F: 5′-CGACGGTCAAAAGTTCCATAAATTC-3′, ETTups-1R: 5′-TAGTGCGACCATAAGCAGATATACC-3′. The *ett-2* allele was identified by amplifying DNA with the primers ett-2-dCAPS-F: 5′-CTCTGGTGATGCTGTGCTTTTCCCTA-3′ and ett-2-dCAPS-R: 5′-CATCATCTCCTCTGTATCAGAGAAACC-3′, followed by cleavage with EcoT14I. *rdr6-11* was identified as described previously [48].

### Histological analyses

Observation of developing embryos, sectioning of leaves and meristems embedded in Technovit resin, and histochemical staining of GUS activity were done as described previously [31]. For trypan blue staining, 15-day-old plants were incubated in 0.5 mg/ml trypan blue, dissolved in phenol/glycerol/lactic acid/water/ethanol (1∶1∶1∶1∶8), in a boiling water bath for 1 min. The tissues were left in staining solution at room temperature for 1 h, cleared in chloral hydrate solution, and examined with an Olympus SZX12 stereomicroscope. For scanning electron microscopy, samples were fixed overnight in Carnoa's solution (1∶3 isoamyl acetate∶ethanol), incubated in 1∶1 and then 3∶1 isoamyl acetate∶ethanol for 15 min each, and finally immersed in isoamyl acetate. The materials were then critical-point-dried in liquid CO_2_, coated with platinum and palladium, and examined with a Hitachi S-3000 scanning electron microscope. For observation of *FILp:GFP*, shoot apices were embedded in 6% agar with 0.05% Silwet L77, and transverse sections of 100–150 µm were obtained using a LinearSlicer Pro 10 (D.S.K.). Sections were mounted with a drop of water and examined using an Olympus FV500 confocal laser scanning microscope. Both GFP and chlorophyll are excited at 488 nm, and the emission was split using a 560 nm dichroic mirror and collected through a 505–525 nm band-pass filter and a 560 nm long-pass filter to observe GFP and chlorophyll, respectively.

### Real time RT–PCR

Total RNA was isolated from 12 to 14-day-old plants that were dissected to separate leaves from shoot apices. Leaves were defined as leaves with recognizable petioles, and shoot apices were defined as the remaining aerial parts. Total RNA was isolated using the RNeasy Plant Mini Kit (Qiagen) according to the manufacturer's instructions. Next, cDNA was synthesized from DNase I-treated total RNA using an oligo(dT) primer and SuperScript III Reverse Transcriptase (Invitrogen). Quantitative real time PCR was carried out using an iCycler iQ system (Bio-Rad) with iQ SYBR Green Supermix (Bio-Rad) as described previously [31]. Primer pairs for each gene were designed to amplify specific fragments of approximately 100 bp. *ARF2*: 5′-TCTTCGATGCTTACCAGAGAAGGTAC-3′ and 5′-ACACTCTACACTCTCAGTATGTTTCG-3′, *ETT*: 5′-CCTGATATCCCTGTCTCTGAG-3′ and 5′-CATCCGAACAAGTGTTGATAAAACC-3′, *ARF4*: 5′-CCGGAAACCCCATAACAAAAAGG-3′ and 5′-TGAGACTGCATCGCAAAATCCAG-3′, *FIL*: 5′-CGTTGGTGTGACTCCTTATTAAAGAG-3′ and 5′-CCACAACTTTTGGACATGATAAACCC-3′, At1g20320: 5′-CTATCACAGGTACTGGAAGTAGAGTG-3′ and 5′-GCCACATTTTACCATATGGAATCTTCG-3′, At1g22930: 5′-GCTTGTGTCTCAGTCAGAGTTCATGG-3′ and 5′-CAAGAGGCTATACAAGTTTACCGAGTG-3′, At1g28120: 5′-CCAAGCCATCTTGTAAGGTATCAGAC-3′ and 5′-CATCCACATGAATCCATATTACCACAG-3′, At1g32460: 5′-CCAAGAAGTTTAAAGGTATTGATGGAAC-3′ and 5′-GTGAAGTGTAGGAGATTTCGATGAGC-3′. The threshold cycles at which fluorescence of the PCR product::SYBR Green complex first exceeded a background level were determined, and the relative template concentrations compared to that of the control were determined based on a standard curve for each gene made using a cDNA dilution series. Relative levels of *ACTIN2* mRNA were used as a reference. The real time PCR assays were performed in duplicate for each cDNA.

### Microarrays

Microarray analysis was done using Affymetrix GeneChip ATH1. Total RNA from shoot apices of 14-day-old plants was analyzed. Replicate experiments were done using different combinations of *teb* and *atr* alleles. In the first experiment, Col-0 (wild-type), *teb-1*, *atr-2*, and *teb-1 atr-2* were used. In the other, Col-0, *teb-2*, *atr-4*, and *teb-2 atr-4* were used. For each sample, 5 µg of total RNA was processed using the GeneChip One-Cycle cDNA Synthesis Kit and the IVT Labeling Kit (Affymetrix) according to the manufacturer's instructions (GeneChip Expression Analysis Technical Manual; Affymetrix) to produce biotin-labeled cRNA. Next, 20 µg of the resulting biotin-labeled cRNA was fragmented to an average strand length of 100 bases (range, 35–200 bases). Subsequently, 15 µg of fragmented cRNA was hybridized to an Affymetrix GeneChip ATH1 and the hybridized chip was washed, stained with streptavidin-phycoerythrin, and scanned. Basic data analysis used to obtain values for signal intensity and detection calls, i.e., ‘present (P)’, ‘marginal (M)’, and ‘absent (A)’, were carried out using GeneChip Operating Software 1.2 (Affymetrix). Further data analysis, including normalization, was performed with GeneSpring GX 7.3 (Agilent Technologies). After values less than 0.01 were set to 0.01, data from each chip were normalized to the 50th percentile of values from that chip. For comparison, the values for each gene were normalized to those of Col-0 by setting values of all genes in Col-0 to 1. Subsequently, we used only a set of genes for which the detection call was ‘P’ or ‘M’ in at least 2 of the 4 samples in each experiment. The raw and normalized data files and details of labeling and hybridization have been deposited in a public microarray database (http://www.ebi.ac.uk/arrayexpress) under accession number E-MEXP-1329. The list of duplicate genes and TAGs has been described previously [52]. Briefly, two datasets were defined as duplicate genes. The high stringency (H) set included protein pairs that share at least 50% identity over 90% of the protein length, whereas the low stringency (L) set included protein pairs with at least 30% identity over 70% of the protein length. TAGs were identified as subsets of duplicate genes. Genes were defined as TAGs if they belonged to the same family of duplicate genes and were physically adjacent. The list of γ-irradiation-induced genes has been described previously [38]. The data for microarray analysis that compares the *sni1* mutant with wild-type Col-0 [54] was obtained from NASCArrays database (http://affymetrix.arabidopsis.info/). For statistical analysis in Figure 5, Figure 6, Figure S6, and Figure S8, we first converted the ratios of expression from linear to logarithmic scale. Then the difference between mean values of two different groups of genes was tested by Student t test.

## Supporting Information

Figure S1 *atm* mutations do not affect the developmental phenotypes of *teb*. Shoot morphology of 2-week-old wild-type (WT), *teb*, *atm*, and *teb atm* double mutant plants. The morphology of *teb atm* cannot be distinguished from that of *teb*. Scale bar, 5 mm.
(2.52 MB TIF)

Figure S2 Cell death phenotype of *teb*, *teb atr*, and *teb atm*. Trypan blue staining to visualize cell death in wild-type (WT) and several single and double mutants. Blue signals indicating cell death are visible in some leaves and cotyledons of *teb-1 atr-2* and *teb-1 atm-4*.
(5.10 MB TIF)

Figure S3 TEB represses *ETT* and *ARF4* in a pathway different from the AGO7- and RDR6-mediated pathway. (A) Rosette phenotypes of 20-day-old wild-type (WT), *ago7-1*, *rdr6-11*, *teb-1*, *teb-1 ago7-1*, *teb-1 rdr6-11* plants. Scale bar, 5 mm. (B) The levels of *ETT* and *ARF4* mRNAs in *teb-1* (t), *ago7-1* (a), *teb-1 ago7-1* (ta), *rdr6-11* (r), and *teb-1 rdr6-11* (tr) relative to wild-type (w) as determined by quantitative real time RT-PCR. The values represent means of 5 biological replicate ±S.E.
(3.10 MB TIF)

Figure S4 Real time RT-PCR analysis of expressions of *Helitron*-flanked genes. The mRNA levels of 4 genes with *Helitron* AtREP3 in their upstream regions in *teb-1* (t), *atr-2* (a), and *teb-1 atr-2* (ta) relative to wild-type (w) as determined by quantitative real time RT-PCR. The values represent means of 5 biological replicate ±S.E.
(0.53 MB TIF)

Figure S5 Summary of microarray analysis. (A) Expression profile for genes with a ‘P’ or ‘M’ call for at least 4 of 8 samples. After per-chip normalization, the values of each gene were normalized to the mean values for the wild-type (WT) in two experiments. Colors represent normalized expression levels for *teb-1 atr-2*, as indicated in the color bar (right). (B) Venn diagram of differentially expressed genes. Magenta circles, genes with more than 1.5-fold higher or lower expression in *teb* than in wild-type; green circles, genes with more than 1.5-fold higher or lower expression in *teb atr* than in wild-type. Yellow, the overlap of these gene groups. Many genes that exhibited higher and lower expression in *teb* than in wild-type also showed higher and lower expression in *teb atr* than in wild-type, respectively. Furthermore, for about three-quarters of the genes in common, the difference in expression was more pronounced in *teb atr* than in *teb*, suggesting that the molecular phenotype of *teb* related to gene expression is enhanced by *atr*.
(3.63 MB TIF)

Figure S6 Biological reproducibility of Figure 5 and Figure 6. Result of second experiment of microarray analysis. Graphs are shown in the same way as in Figure 5 and Figure 6.
(1.26 MB TIF)

Figure S7 *teb* and *teb atr* activate DSB-inducible genes. (A) Expression profile for genes annotated as increased (I) or marginally increased (MI) after γ-irradiation (ionizing radiation; IR) in two experiments (for details, see [38]), and with a ‘P’ or ‘M’ call in at least 4 of 8 samples in our microarray experiments (1,012 genes). (B) Expression profile for genes with expression levels that increased more than a 5-fold after IR and, with a ‘P’ or ‘M’ call in at least 4 of 8 samples in our microarray experiments (114 genes). Colors in (A) and (B) represent normalized expression levels for *teb-1 atr-2*, as indicated in the color bar on the right. Many genes that are inducible by IR displayed higher expression in *teb* and *teb atr* than in wild-type (WT) and *atr*. (C–H) Histograms showing number of genes with altered expression ratios in pairwise comparisons of *teb*/WT (C, D), *teb atr*/WT (E, F), and *teb atr*/*teb* (G, H). (C), (E), and (G) show the frequency distributions of occurrence of genes with expression that was I or MI after IR (1089 genes), and (D), (F), and (H) show frequency distributions of occurrence of genes with expression levels that increased more than 5-fold after IR (124 genes). Bars indicate the mean values from two experiments. Closed circles, first experiment; open circles, second experiment. Red bars indicate subsets of genes with ratios greater than 1 (showing increased expression), yellow bars indicate subsets with ratios less than 1 (showing decreased expression), and gray bars indicate the subset of genes with detection call ‘A’ for more than 2 of the 4 samples in each experiment. Numbers in graph of (C) indicate the ratios of expression that define each subset.
(4.29 MB TIF)

Figure S8 Upregulation of TAGs in *sni1*. Frequency distribution histograms of *sni1*/WT ratios of expression for all genes with ‘P’ call for at least 1 of 2 samples in each experiment. Distribution of TAGs H (magenta lines) and other genes (blue lines) are shown. Results from 3 independent experiments are shown.
(0.41 MB TIF)

Figure S9 Upregulation of *PR* genes in *teb* and *teb atr*. The levels of *PR1*, *PR2*, and *PR5* mRNAs in wild-type (WT), *teb*, *atr*, and *teb atr*, as determined by microarrays. The values are expressed as the ratio to the value obtained for the wild-type in each experiment. Bars indicate the mean values from two experiments. Closed circles, first experiment; open circles, second experiment.
(0.30 MB TIF)
