# Supplementary figures and images for: A Link among DNA Replication, Recombination, and Gene Expression Revealed by Genetic and Genomic Analysis of TEBICHI Gene of Arabidopsis thaliana
Source: PLoS Genet. 2009 Aug 21;5(8):e1000613. doi: 10.1371/journal.pgen.1000613 (PMC2721414; doi:10.1371/journal.pgen.1000613)

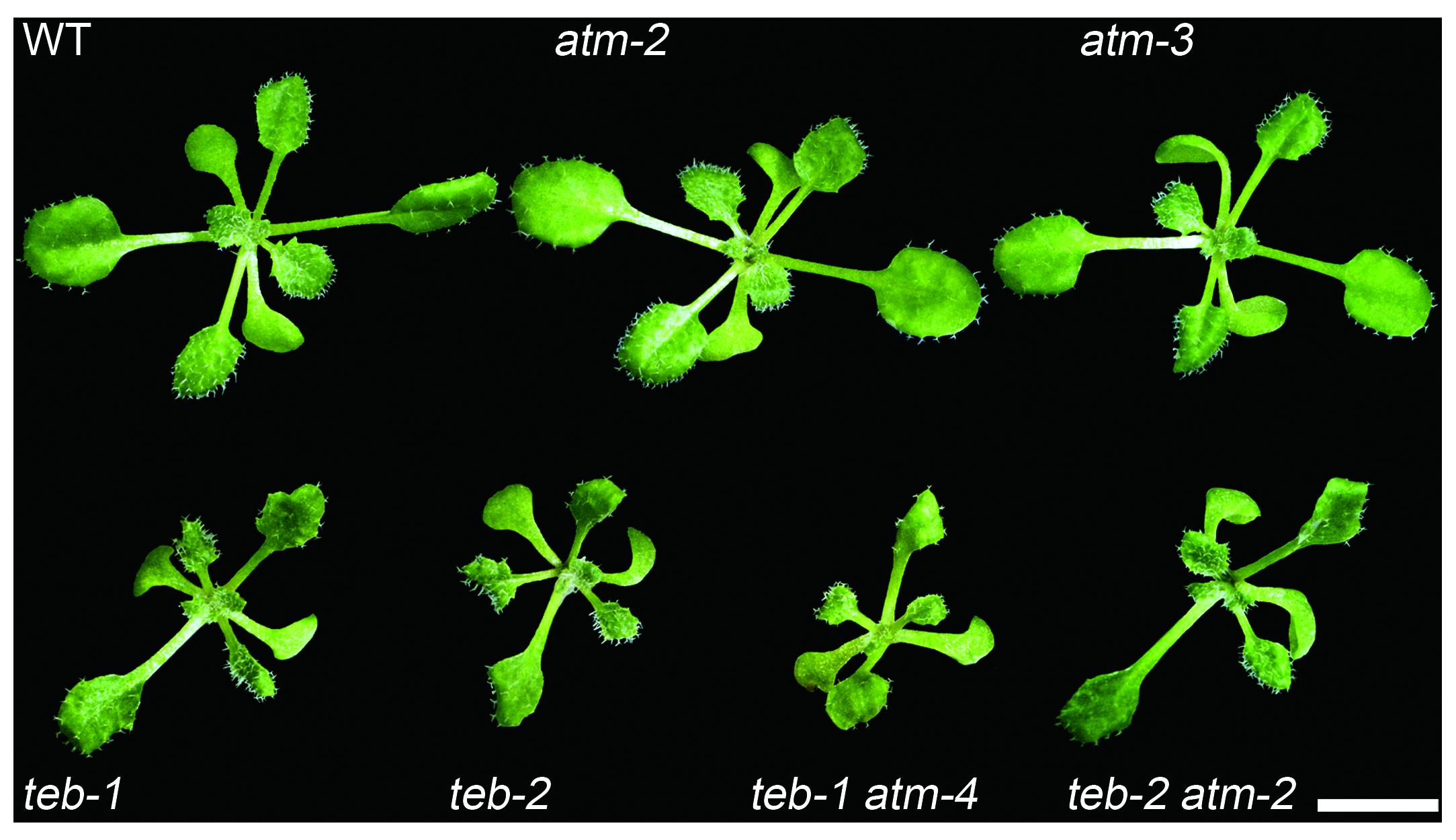

Supplement: Figure S1 — atm mutations do not affect the developmental phenotypes of teb. Shoot morphology of 2-week-old wild-type (WT), teb, atm, and teb atm double mutant plants. The morphology of teb atm cannot be distinguished from that of teb. Scale bar, 5 mm. (2.52 MB TIF) [file pgen.1000613.s001.tif]

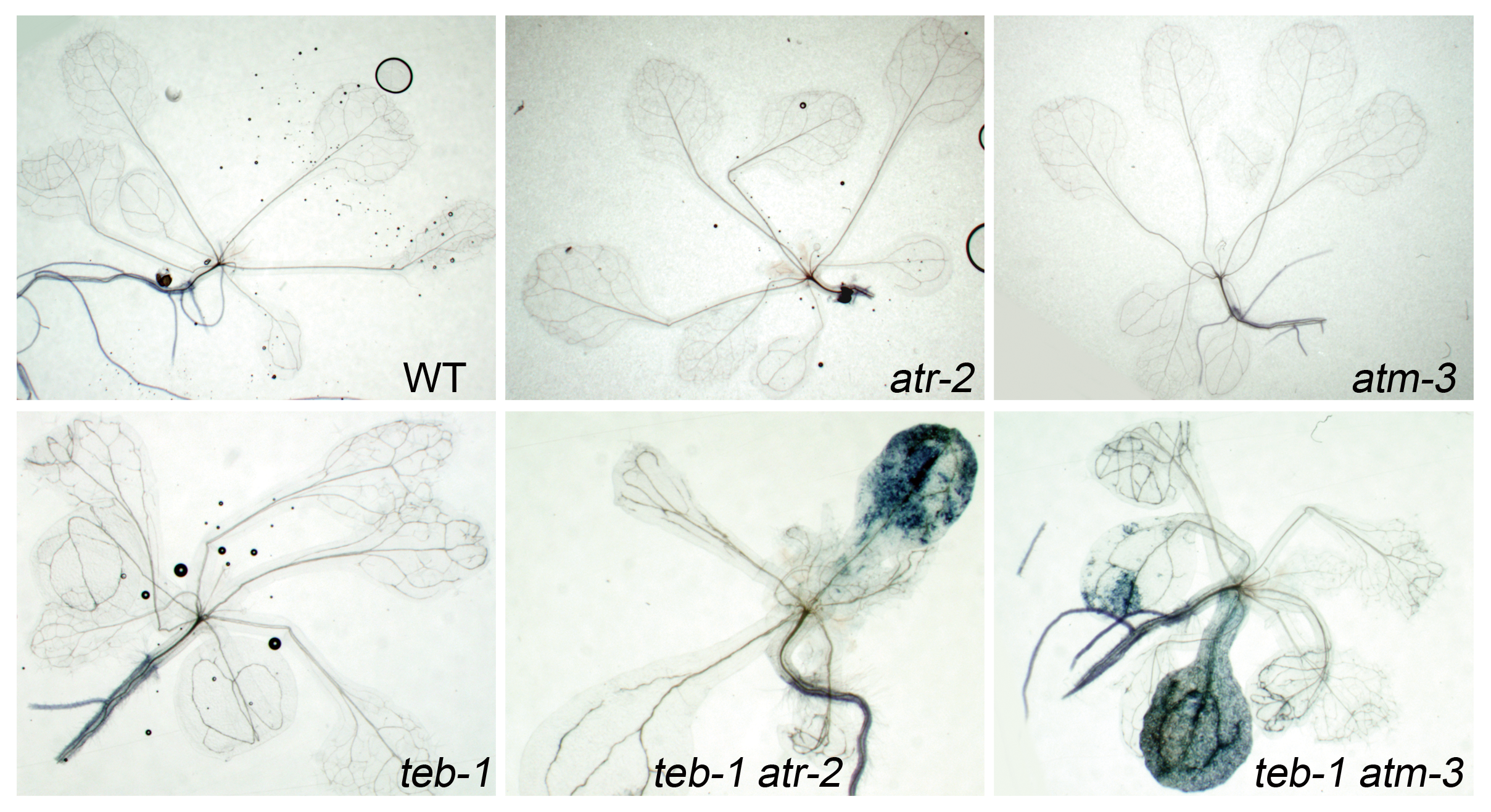

Supplement: Figure S2 — Cell death phenotype of teb, teb atr, and teb atm. Trypan blue staining to visualize cell death in wild-type (WT) and several single and double mutants. Blue signals indicating cell death are visible in some leaves and cotyledons of teb-1 atr-2 and teb-1 atm-4. (5.10 MB TIF) [file pgen.1000613.s002.tif]

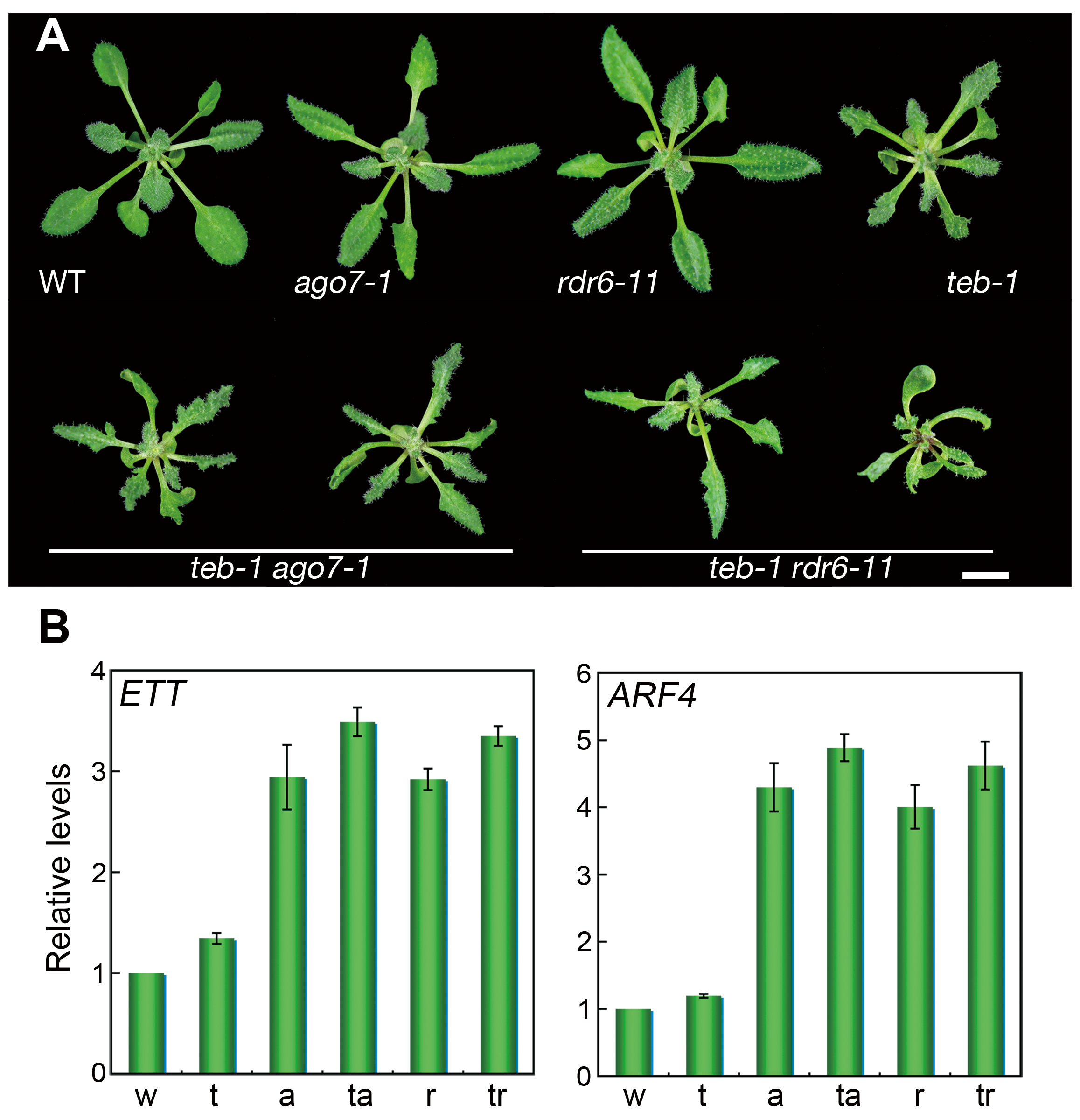

Supplement: Figure S3 — TEB represses ETT and ARF4 in a pathway different from the AGO7- and RDR6-mediated pathway. (A) Rosette phenotypes of 20-day-old wild-type (WT), ago7-1, rdr6-11, teb-1, teb-1 ago7-1, teb-1 rdr6-11 plants. Scale bar, 5 mm. (B) The levels of ETT and ARF4 mRNAs in teb-1 (t), ago7-1 (a), teb-1 ago7-1 (ta), rdr6-11 (r), and teb-1 rdr6-11 (tr) relative to wild-type (w) as determined by quantitative real time RT-PCR. The values represent means of 5 biological replicate ±S.E. (3.10 MB TIF) [file pgen.1000613.s003.tif]

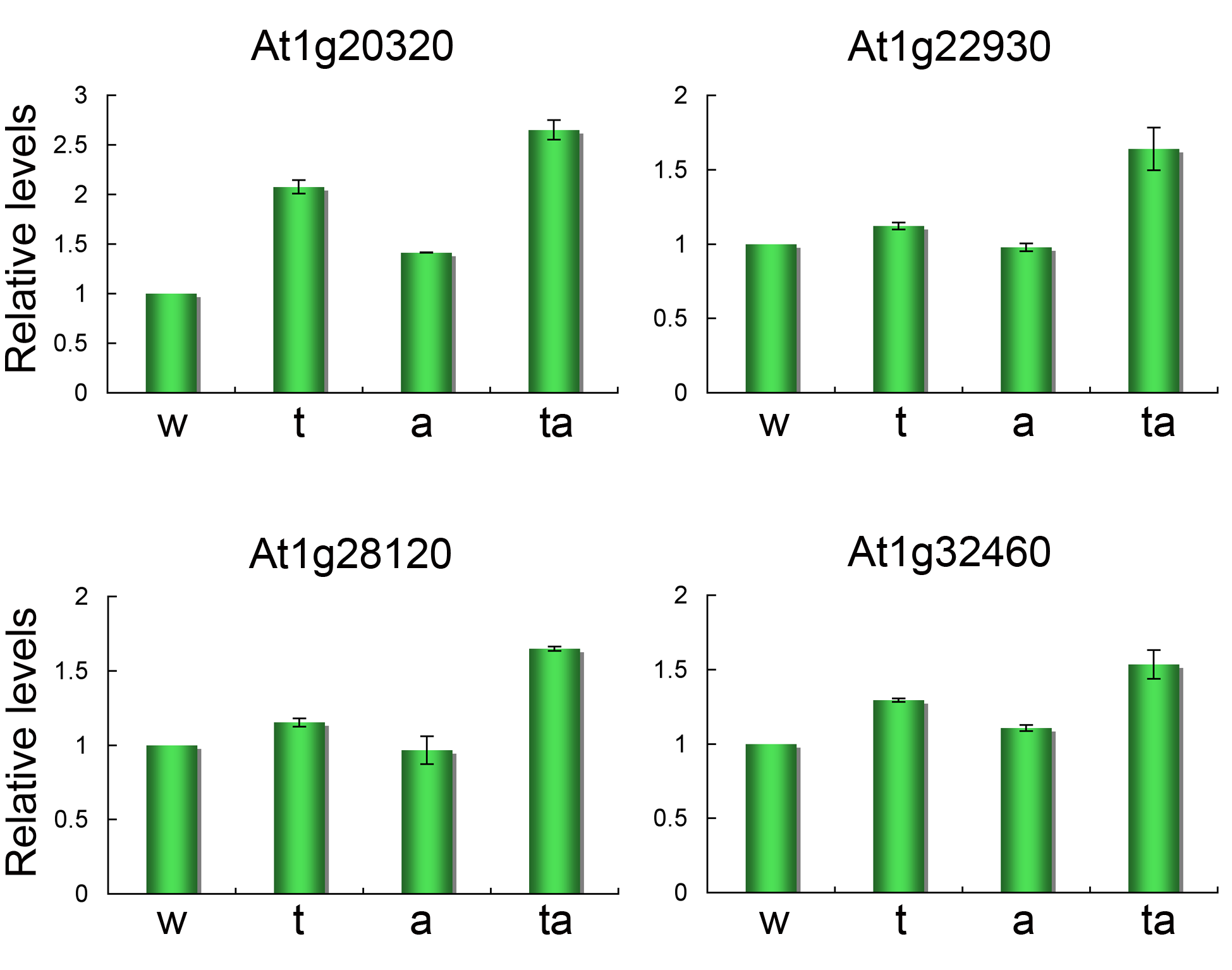

Supplement: Figure S4 — Real time RT-PCR analysis of expressions of Helitron-flanked genes. The mRNA levels of 4 genes with Helitron AtREP3 in their upstream regions in teb-1 (t), atr-2 (a), and teb-1 atr-2 (ta) relative to wild-type (w) as determined by quantitative real time RT-PCR. The values represent means of 5 biological replicate ±S.E. (0.53 MB TIF) [file pgen.1000613.s004.tif]

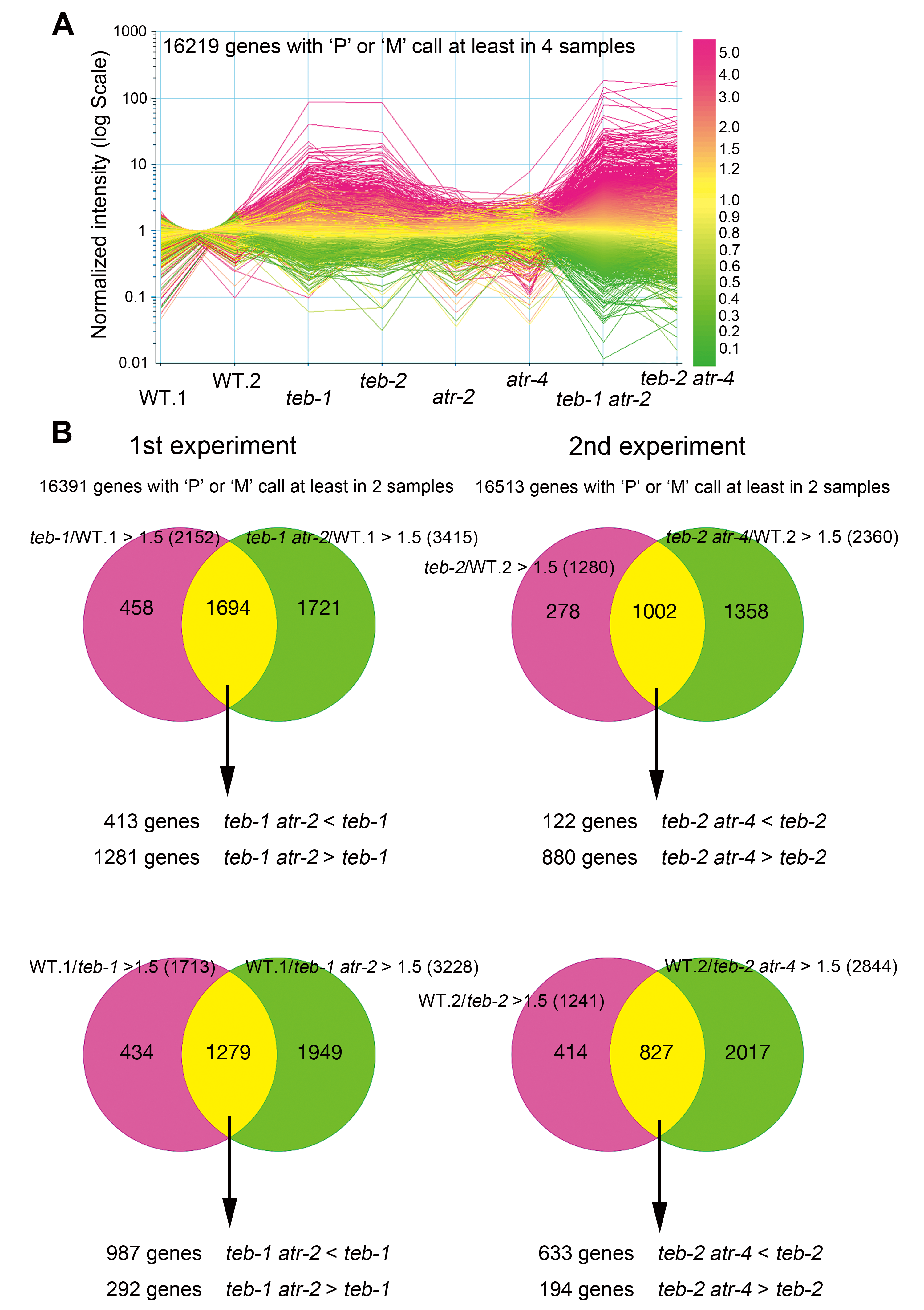

Supplement: Figure S5 — Summary of microarray analysis. (A) Expression profile for genes with a ‘P’ or ‘M’ call for at least 4 of 8 samples. After per-chip normalization, the values of each gene were normalized to the mean values for the wild-type (WT) in two experiments. Colors represent normalized expression levels for teb-1 atr-2, as indicated in the color bar (right). (B) Venn diagram of differentially expressed genes. Magenta circles, genes with more than 1.5-fold higher or lower expression in teb than in wild-type; green circles, genes with more than 1.5-fold higher or lower expression in teb atr than in wild-type. Yellow, the overlap of these gene groups. Many genes that exhibited higher and lower expression in teb than in wild-type also showed higher and lower expression in teb atr than in wild-type, respectively. Furthermore, for about three-quarters of the genes in common, the difference in expression was more pronounced in teb atr than in teb, suggesting that the molecular phenotype of teb related to gene expression is enhanced by atr. (3.63 MB TIF) [file pgen.1000613.s005.tif]

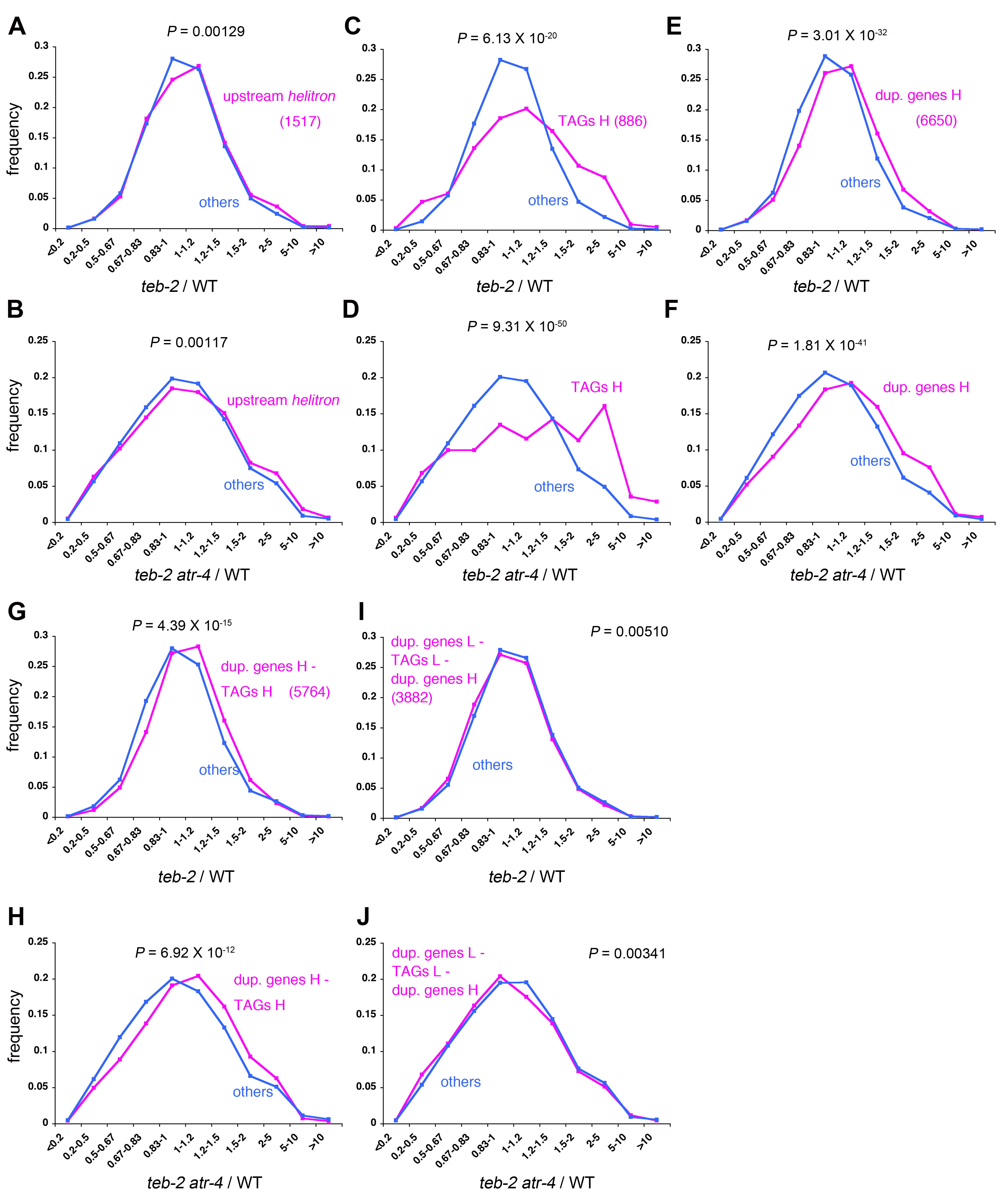

Supplement: Figure S6 — Biological reproducibility of Figure 5 and Figure 6. Result of second experiment of microarray analysis. Graphs are shown in the same way as in Figure 5 and Figure 6. (1.26 MB TIF) [file pgen.1000613.s006.tif]

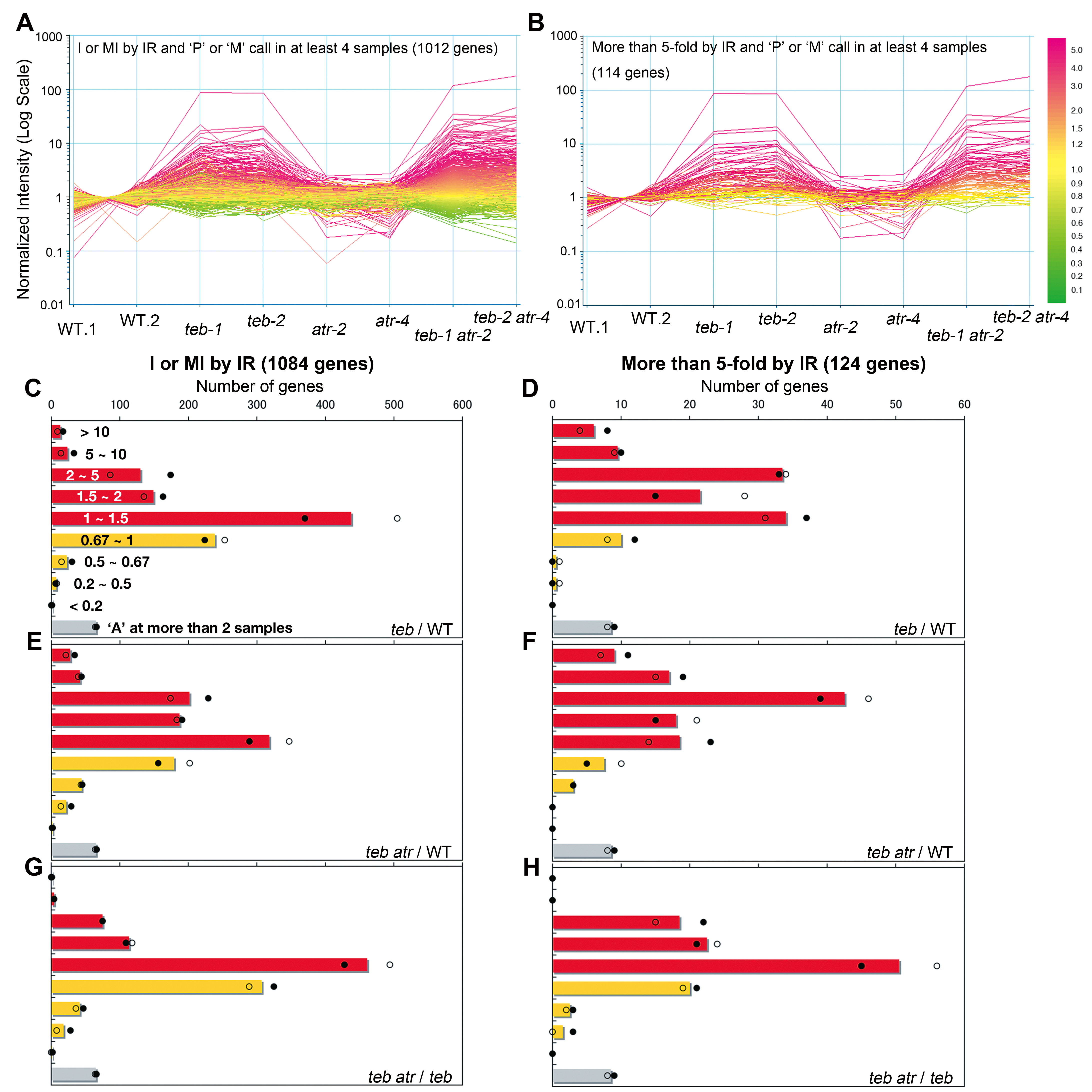

Supplement: Figure S7 — teb and teb atr activate DSB-inducible genes. (A) Expression profile for genes annotated as increased (I) or marginally increased (MI) after γ-irradiation (ionizing radiation; IR) in two experiments (for details, see [38]), and with a ‘P’ or ‘M’ call in at least 4 of 8 samples in our microarray experiments (1,012 genes). (B) Expression profile for genes with expression levels that increased more than a 5-fold after IR and, with a ‘P’ or ‘M’ call in at least 4 of 8 samples in our microarray experiments (114 genes). Colors in (A) and (B) represent normalized expression levels for teb-1 atr-2, as indicated in the color bar on the right. Many genes that are inducible by IR displayed higher expression in teb and teb atr than in wild-type (WT) and atr. (C–H) Histograms showing number of genes with altered expression ratios in pairwise comparisons of teb/WT (C, D), teb atr/WT (E, F), and teb atr/teb (G, H). (C), (E), and (G) show the frequency distributions of occurrence of genes with expression that was I or MI after IR (1089 genes), and (D), (F), and (H) show frequency distributions of occurrence of genes with expression levels that increased more than 5-fold after IR (124 genes). Bars indicate the mean values from two experiments. Closed circles, first experiment; open circles, second experiment. Red bars indicate subsets of genes with ratios greater than 1 (showing increased expression), yellow bars indicate subsets with ratios less than 1 (showing decreased expression), and gray bars indicate the subset of genes with detection call ‘A’ for more than 2 of the 4 samples in each experiment. Numbers in graph of (C) indicate the ratios of expression that define each subset. (4.29 MB TIF) [file pgen.1000613.s007.tif]

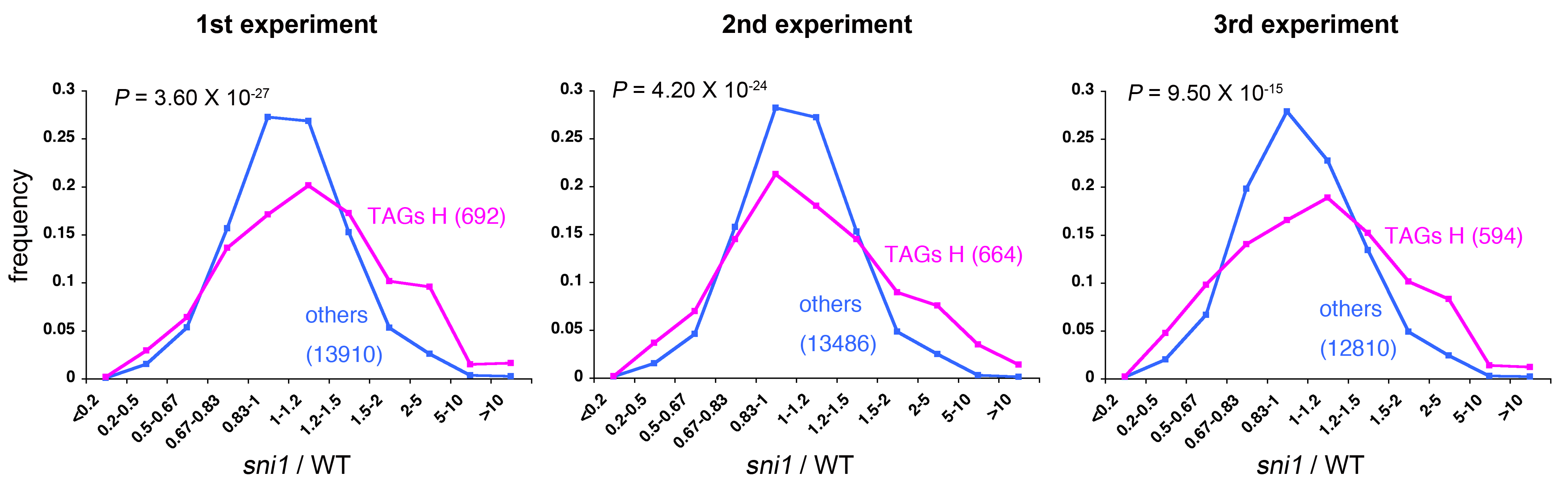

Supplement: Figure S8 — Upregulation of TAGs in sni1. Frequency distribution histograms of sni1/WT ratios of expression for all genes with ‘P’ call for at least 1 of 2 samples in each experiment. Distribution of TAGs H (magenta lines) and other genes (blue lines) are shown. Results from 3 independent experiments are shown. (0.41 MB TIF) [file pgen.1000613.s008.tif]

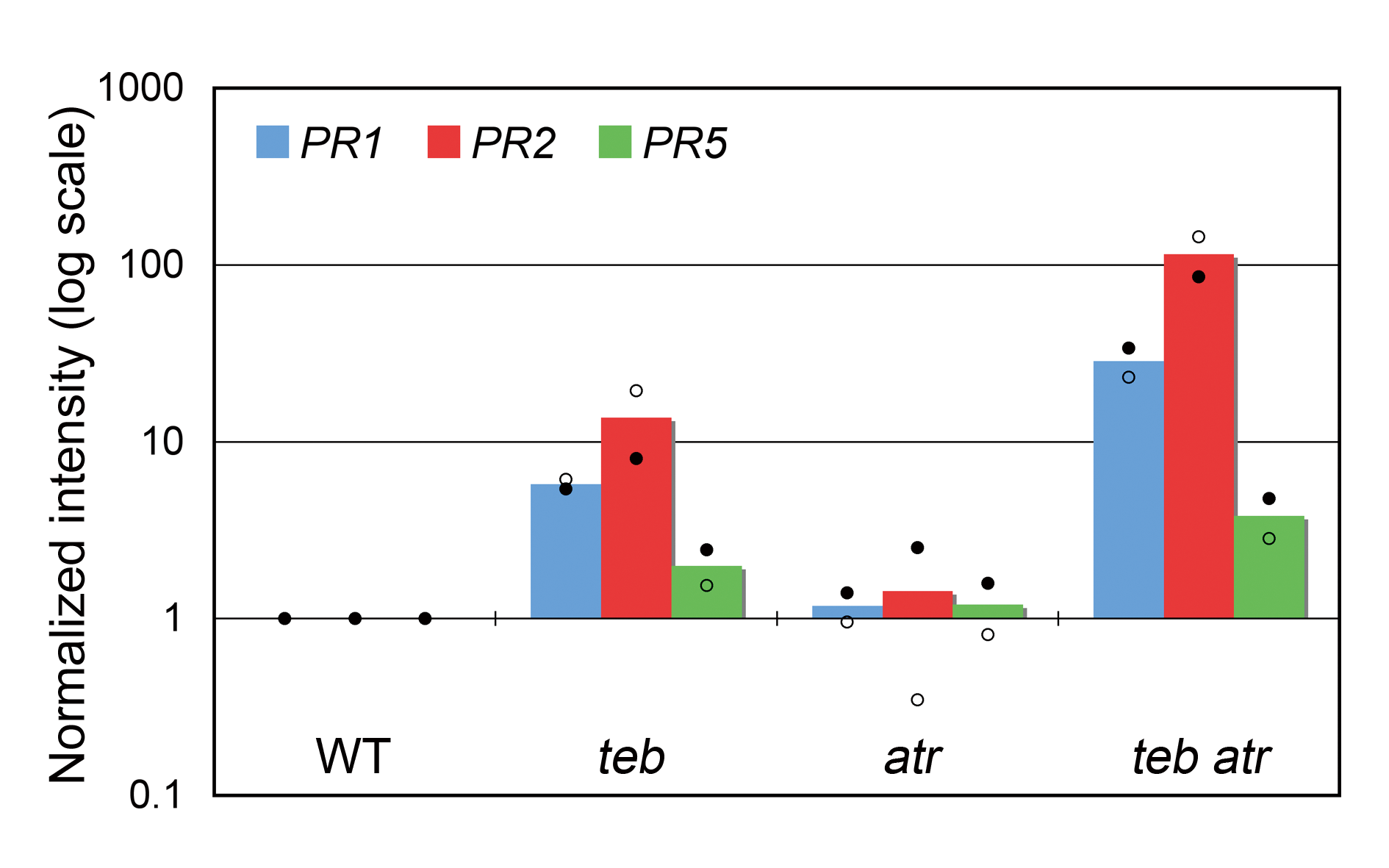

Supplement: Figure S9 — Upregulation of PR genes in teb and teb atr. The levels of PR1, PR2, and PR5 mRNAs in wild-type (WT), teb, atr, and teb atr, as determined by microarrays. The values are expressed as the ratio to the value obtained for the wild-type in each experiment. Bars indicate the mean values from two experiments. Closed circles, first experiment; open circles, second experiment. (0.30 MB TIF) [file pgen.1000613.s009.tif]
